# Co-Delivery of *p53* Restored and *E7* Targeted Nucleic Acids by Poly (Beta-Amino Ester) Complex Nanoparticles for the Treatment of HPV Related Cervical Lesions

**DOI:** 10.3389/fphar.2022.826771

**Published:** 2022-02-04

**Authors:** Jinfeng Xiong, Guannan Li, Xinyu Mei, Jiahui Ding, Hui Shen, Da Zhu, Hui Wang

**Affiliations:** ^1^ Department of Obstetrics and Gynecology, Tongji Hospital, Tongji Medical College, Huazhong University of Science and Technology, Wuhan, China; ^2^ School of Pharmacy, Tongji Medical College, Huazhong University of Science and Technology, Wuhan, China

**Keywords:** p53, human papillomavirus, cervical cancer, poly (beta-amino ester), nanoparticle

## Abstract

The *p53* gene has the highest mutation frequency in tumors, and its inactivation can lead to malignant transformation, such as cell cycle arrest and apoptotic inhibition. Persistent high-risk human papillomavirus (HR-HPV) infection is the leading cause of cervical cancer. *P53* was inactivated by HPV oncoprotein *E6*, promoting abnormal cell proliferation and carcinogenesis. To study the treatment of cervical intraepithelial neoplasia (CIN) and cervical cancer by restoring *p53* expression and inactivating HPV oncoprotein, and to verify the effectiveness of nano drugs based on nucleic acid delivery in cancer treatment, we developed poly (beta-amino ester)537, to form biocompatible and degradable nanoparticles with plasmids (expressing *p53* and targeting *E7*). *In vitro* and *in vivo* experiments show that nanoparticles have low toxicity and high transfection efficiency. Nanoparticles inhibited the growth of xenograft tumors and successfully reversed HPV transgenic mice’s cervical intraepithelial neoplasia. Our work suggests that the restoration of *p53* expression and the inactivation of HPV16 *E7* are essential for blocking the development of cervical cancer. This study provides new insights into the precise treatment of HPV-related cervical lesions.

## Background

*P53*, the most commonly mutated gene in human cancer, is essential for maintaining the stability of the human genome (Blandino and Di Agostino, 2018; Mendiratta et al., 2021). Its loss is a key event in the development of various tumors (Klco and Mullighan, 2021; Rudin et al., 2021). *P53* plays an important role in protecting cells from malignant transformation by inducing cell cycle arrest or apoptosis (Zhou et al., 2019). In recent years, researchers have tried to reactivate inactivated *p5*3. In spontaneous *p53* mutant lymphoma and sarcoma models, the restored expression of wild-type *p53* resulted in tumor growth arrest (Wang et al., 2011). Activating *p53*-dependent apoptosis in zebrafish inhibited angiogenesis (Zhou et al., 2016). Restoring *p53* expression induced cycle arrest and apoptosis in non-small cell lung cancer (NSCLC) (Lu et al., 2017). Therefore, restoring *p53* expression is a promising method in a variety of tumors.

Cervical cancer is the second most common cancer in less developed regions (Ferlay et al., 2015). Each year, 570,000 women are diagnosed with cervical cancer and 311,000 women die from this disease, which seriously affects women’s health worldwide (Bray et al., 2018; Arbyn et al., 2020). Persistent high-risk human papillomavirus (HR-HPV) infection is the leading cause of cervical cancer (Francis et al., 2010). Oncoproteins *E6* and *E7* are key factors in HPV-associated carcinogenesis. *P53* was also inactivated by *E6*, promoting abnormal cell proliferation and carcinogenesis (Moody and Laimins, 2010; Taghizadeh et al., 2019). The HPV oncogenes *E6* and *E7* and their related host genes (such as *p53*) are key targets for prevention and treatment in the long process from HPV infection to CIN and ultimately cervical cancer (Pal and Kundu, 2019).

To date, surgical treatment is mainly used for CIN and early cervical cancer, however, it can’t solve the problem of virus infection and subsequent changes in key cell-signaling pathways. In recent years, an increasing number of gene-targeted therapies based on recombinant plasmids have been reported (Dominguez et al., 2016; Evers et al., 2016; Pahle and Walther, 2016; Zhang et al., 2016; Almeida et al., 2019). The CRISPR/Cas9 system is a powerful tool for the prevention and treatment of HPV infection due to its simple composition, convenient operation, high mutation efficiency, and low cost (Chen et al., 2019; Sharma et al., 2021; Song et al., 2021). Furthermore, it is necessary to develop a suitable vector to ensure the successful delivery of recombinant plasmids (Xiao et al., 2014; Xu et al., 2021). Clinical applications of viral vectors are limited due to safety issues (Zhou et al., 2017; Li et al., 2021; Ma et al., 2021). Non-viral vectors such as cationic liposomes (Wang et al., 2021), polyethyleneimine (PEI) (Radmanesh et al., 2021), and poly (β-amino ester) (PBAE) (Zhu et al., 2018; Zhang et al., 2021) are widely used due to their low toxicity and nonimmune characteristics. PBAEs have good biocompatibility and biodegradability. These materials can compress plasmids into nanosized composite particles, protect plasmids from nuclease degradation, and effectively deliver plasmids to cells (Tzeng et al., 2013; Guerrero-Cázares et al., 2014; Kim et al., 2016; Kaczmarek et al., 2021).

Therefore, to study the treatment of CIN and cervical cancer by restoring p53 expression and inactivating HPV oncoprotein, and to verify the effectiveness of nano drugs based on nucleic acid delivery in cancer treatment, we attempted to reverse cervical intraepithelial neoplasia by vaginal injection of nanoparticles composed of PBAE537 and plasmids (wild-type *p53* expression and HPV16 *E7* targeting), providing a new idea strategy for precise treatment of cervical cancer.

## Methods

### Materials and Synthesis of Poly (Beta-Amino ester)537

**FIGURE 1:**
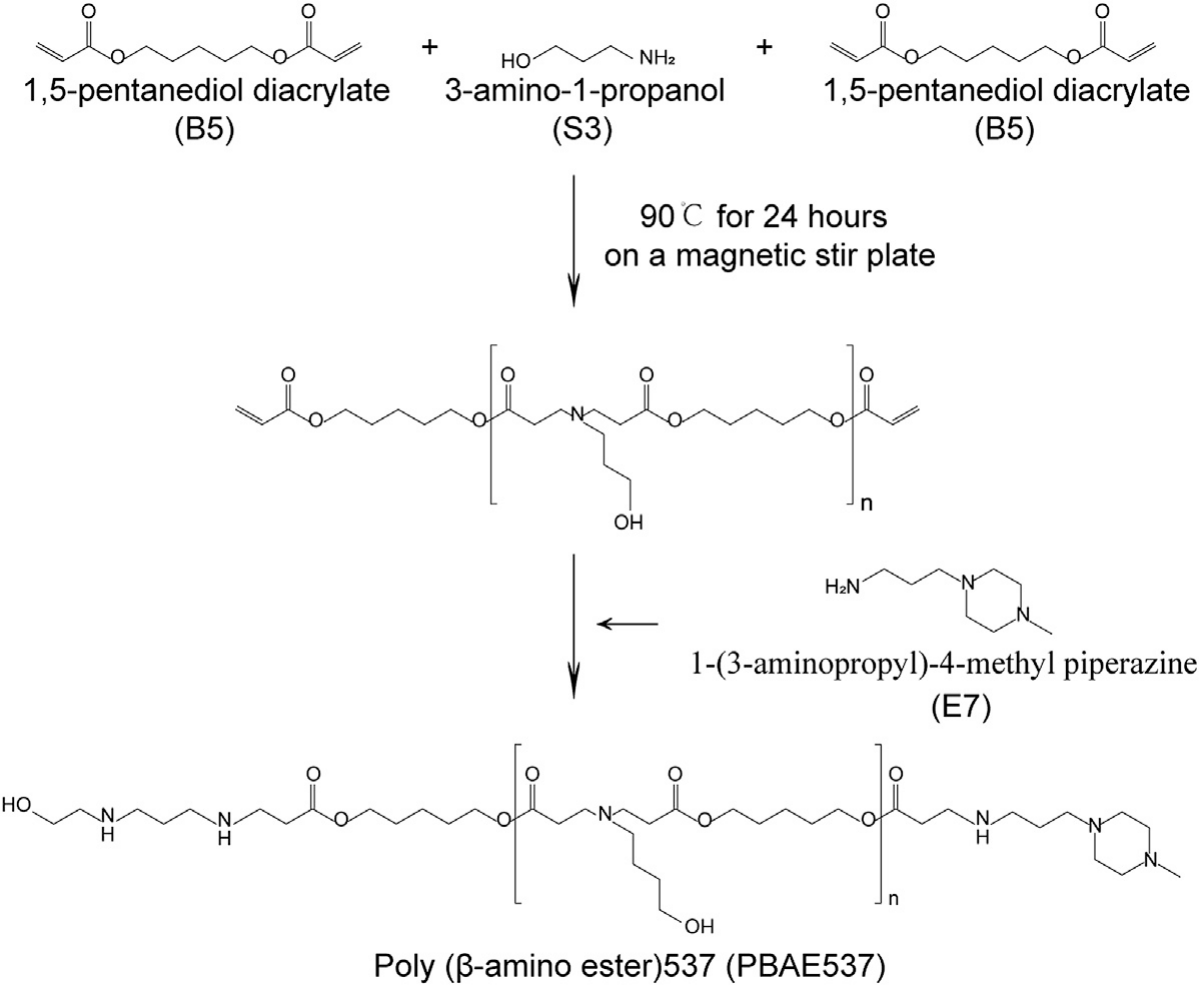
Synthesis of PBAE537. Synthesis of PBAE537. The PBAE nanomaterial PBAE537 was formed by mixing 1,5-pentanediol diacrylate (B5) with 3-amino-1-propanol (S3) and stirring at 90°C for 24 h on a magnetic stirring plate and adding 1-(3-aminopropyl)-4-methyl piperazine (E7).

We purchased 1,5-pentanediol diacrylate (B5) from Aladdin (B136168, N184580, China), 3-amino-1-propanol (S3) from TCI (A0438, Japan), and 1-(3-aminopropyl)-4-methylpiperazine (E7) from ALFA Aesar (L04876, Ward Hill, MA). Branched poly(ethyleneimine) (water-free, 25 kDa, bPEI) was purchased from Sigma-Aldrich (408727, USA). Cationic liposome HP was purchased from Roche (6366236, USA). The overexpression (OE) *p53* (PCDH-CMV-MCS-EF1-RFP-T2A-Puro) plasmids contained CMV promoter-expressing human *p53* or mouse *p53*. The plasmids pDest-EGFP-N1 (plasmid 31796, GFP) and pAAV-CAG-RFP (plasmid 22910, RFP) were purchased from Addgene. The HPV16 *E7*-targeted CRISPR plasmid sequence was ATA​TTG​TAA​TGG​GCT​CTG​TC CGG. Plasmid DNA was prepared using an endotoxin-free plasmid extraction kit (Omega, USA) and stored at −80°C. PBAE537 was synthesized in a 2-step procedure (Figure 1) as follows: 1,5-pentanediol diacrylate (B5) was mixed with 3-amino-1-propanol (S3) and stirred on a magnetic stir plate at 90°C for 24 h with 2 ml of DMSO at a 1.2:1 M ratio. After stirring, 1-(3-aminopropyl)-4-methyl piperazine (E7) was added to the mixture (10-fold), and the mixture was stirred for 30 s, incubated at room temperature for 1 h, and precipitated in anhydrous diethyl ether to remove the solvent and monomers. The polymer was washed three times with ether and kept under a vacuum with desiccant for 48 h to remove the final traces of ether. The polymer poly (beta-amino ester) 537 was then divided into smaller volumes and stored at −20°C with desiccant until needed. For further use, the polymer was dissolved in DMSO at 100 mg/ml and stored at −20°C.

### Preparation and Characterization of Poly (Beta-Amino ester)537 and Plasmid Polyplex Nanoparticles

PBAE537 (2 μg/L) was diluted with 25 mM sodium acetate (pH 5) into 200 ng of plasmid at mass ratios of 5:1, 10:1, 20:1, 40:1, 60:1, 80:1, and 100:1. PBAE537 and plasmid were mixed gently in 20 µL of NaAc for 30 s and incubated at room temperature for at least 20 min. The particle size and zeta potential of the nanoparticle (NP) were measured by laser light scattering (DB-525 Zeta PALS; Brookhaven Instruments, Holtsville, NY, USA).

### Transmission Electron Microscopy Imaging

Nanoparticles composed of PBAE537/GFP expressing plasmid (hereinafter referred to as the GFP) were deposited on porous carbon film carbon-coated copper mesh and characterized by TEM (JEM-1230, Japan).

### Cells and Animals

The cervical cancer cell lines SiHa, HeLa, CaSki, MS751, C33A, and HEK293 were purchased from ATCC and passaged in our laboratory. The S12 cell line is an immortalized human cervical keratinocyte cell line, and it was a generous gift from Professor Kenneth Raj (Health Protection Agency) with permission from the original owner, Professor Margaret Stanley (Bechtold et al., 2003). SiHa, HeLa, MS751, C33A, and HEK293 cells were cultured in DMEM supplemented with 10% fetal bovine serum (FBS). CaSki cells were grown in RPMI-1640 supplemented with 10% FBS. S12 cells were maintained in a 1:1 mixture of DMEM/F12 (Gibco) and Ham’s F12 (Gibco) medium supplemented with 10% FBS, 24.3 mg/ml adenine, 0.5 mg/ml hydrocortisone, 8.4 ng/ml cholera toxin, 5 mg/ml insulin, and 10 ng/ml epidermal growth factor (EGF). All cells were cultured in a humidified incubator with 5% CO_2_ at 37°C.

C57BL/6 female mice were purchased from Beijing HFK Bioscience, and K14-HPV16 transgenic mice were provided by the National Cancer Institute (NCI) Mouse Repository (Frederick, MD, USA). All mice were housed at the SPF animal laboratory in the Experimental Animal Center, Tongji Medical College, Huazhong University of Science and Technology (HUST, Wuhan, China) and managed by regulations of Chinese law. The research was approved by the Experimental Animal Ethics Committee of Tongji Medical College of Huazhong University of Science and Technology.

### Biocompatibility of Nanoparticles *In Vitro* and *In Vivo*

PBAE537 solution (diluted with 25 mM sodium acetate at pH 5) was added to the green fluorescent protein (GFP) plasmid at mass ratios of 20:1, 40:1, 60:1, and 80:1. SiHa, HeLa, CaSki, and S12 cells were inoculated in 96-well plates with the same number of cells per cell line in each well. Each well was transfected with 100 ng GFP. bPEI was diluted with PBS to 1 mg/mL as a positive control. The cells were treated with nanoparticles for 4 h and then cultured in a complete medium. Cell viability was defined as the metabolic activity retained in each well after transfection, measured at various time points using a Cell Counting Kit-8 (CCK-8, Dojindo) according to the manufacturer’s instructions.

One hundred microliters of nanoparticles (PBAE537/GFP 60:1, 100 µg plasmid) were injected into the thigh muscles of C57BL/6 mice once a day for 3 days. The same volume of bPEI/GFP (bPEI/GFP 3:1, 100 μg) solution was used as a positive control. Thigh muscles and other organs were collected on the 4th and 7th days after the initial injection. PBAE537/GFP or bPEI/GFP (20 µL NPs containing 10 μg GFP) solution was pipetted into the vaginas of C57BL/6 mice once a day for 20 days. The cervix and other organs were then collected and fixed with paraformaldehyde. Toxicity was evaluated by hematoxylin-eosin (H&E) staining.

### Transfection Efficiency

All cells were inoculated in sterile 6-well plates at a density of 40–70% at transfection. The nanoparticles formed by PBAE537 and GFP at different mass ratios (20:1, 40:1, 60:1, 80:1) were dissolved in 100 μL of 25 mM pH 5 sodium acetate and incubated at room temperature for 20 min before being added to plate wells containing 900 μL of serum-free medium. After incubation for 4 h, the nanoparticles were replaced with a complete medium. The bPEI/GFP complex (mass ratio 3:1, 2 μg GFP) was mixed in PBS for 20 min and then added to the cells.

Four-week-old C57BL/6 mice were injected with 20 μL of nanoparticles into the vagina once a day for 3 days. The vaginas were rinsed with PBS 3 times before each administration to remove vaginal mucus. Three days after administration, the mice were euthanized, and the cervix was separated and frozen into 7 μm sections using Thermo Fisher Scientific.

### Xenograft Experiments

SiHa cells (5×10^6^) were injected subcutaneously into 4-week-old nude mice. When the tumor grew to nearly 35 mm^3^, the mice were randomly assigned to four groups. We administered intra-tumoral injections of 100 μL of NPs coated with 60 μg of plasmid (PBAE537/plasmid 60:1) every 4 days. Subcutaneous tumors were collected after the mice were euthanized, and the tumor size was calculated using the following formula: L × W (Blandino and Di Agostino, 2018) × 0.5. All experimental protocols were approved by the Institutional Animal Care and Use Committee of HUST, and the study was carried out in strict accordance with the Guidelines for the Welfare of Animals in Experimental Neoplasia.

### Transgenic Mouse Experiments

Six-week-old K14-HPV16 transgenic female mice were randomly divided into four groups for vaginal administration. After anesthesia with 4% chloral hydrate, the vagina was irrigated 3 times with PBS. PBAE537 and 10 μg plasmid with a final volume of 20 μL (PBAE537/plasmid 60:1) were injected into the mouse vaginas once a day for 20 days. The drugged mice were placed in the supine position for at least 20 min while anesthetized to prevent the drug from flowing out of the vagina due to pressure. After 20 days of treatment, the mice were euthanized, and the cervix was dissected and fixed with 4% paraformaldehyde. The cervical tissues of the mice were paraffin-embedded, sectionalized, and stained with HE and IHC staining.

### Immunohistochemical Staining

Paraffin-embedded sections (5 μm) were stained with H and E and immunohistochemical (IHC) staining. The slides were incubated overnight at 4°C with mouse anti-HPV16 *E7* (1:50, orb10573, Biorbyt), rabbit anti-*P16* (1:100, A0262, Abclonal), rabbit anti-*RB1* (1:100, 10048-2-Ig, Proteintech), rabbit anti-*Ki67* (1:100, ab16667, Abcam), rabbit anti-*CDK2* (1:400, ab6538, Abcam), rabbit anti-*E2F1* (1:200,12171-1-Ap, Proteintech), rabbit anti-*TP53* (1:150, A16989, Abclonal), rabbit anti-*14-3-3 Sigma* (1:50, A1026, Abclonal) and rabbit anti-*P21* (1:150, A11454, Abclonal) primary antibodies. Diaminobenzidine (DAB) was used for antibody detection. Images were photographed from three randomly selected fields using cellSens Dimension (version 1.8.1, Olympus). Staining was assigned a score using a semiquantitative six category grading system: 0, no staining; 1,1%to 10% staining; 2, 11% to 25% staining; 3, 26% to 50% staining; 4, 51% to 75% staining; and 5, >75% staining. Staining intensity was assigned a score using a semiquantitative four-category grading system: 0, no staining; 1, weak staining; 2, moderate staining; and 3, strong staining. Every core was assessed individually and the mean of three readings was calculated for every slide. The staining score was determined separately by two experts under the same conditions.

### Western Blot

Cells were lysed on ice for 30 min in lysis buffer containing 150 mmol/L NaCl, 1% sodium deoxycholate, 50 mmol/L Tris, 1% Triton X-100, 0.1% SDS, and a protease inhibitor cocktail. The primary antibodies used were rabbit anti-GAPDH (1:1,000, AM1020a, Abgent) and mouse anti-Cas9 (1:200, 14,697, CST).

### Statistical Analysis

All quantitative data are expressed as the mean ± SEM of at least three parallel measurements. One-way ANOVA was used for statistical analysis, and GraphPad Prism software was used for *p* < 0.05.

## Results

### Synthesis and Characterization of Nanoparticles

**FIGURE 2:**
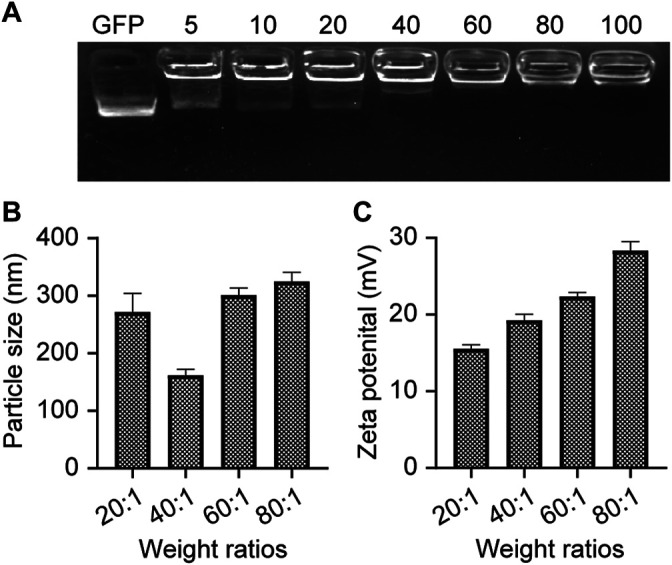
Characterization of NPs. **(A)** Agarose gel (2%) electrophoresis of NPs composed of PBAE537 and GFP at different mass ratios. **(B)** Measurement of particle sizes of NPs with different mass ratios (PBAE537:GFP) and **(C)** zeta potentials by dynamic light scattering. The data represent the mean ± SEM (*n* = 3 per group).

Poly β-amino ester 537 (PBAE537) was synthesized from 1,5-pentanediol diacrylate (B5), 3-amino-1-propanol (S3) and 1-(3-amino-propyl)-4-methyl piperazine (E7) as monomers. The polymer was named PBAE537 according to the three monomers of the main chain (B), side chain (S), and end cap (E). The synthetic scheme of PBAE537 is shown in Figure 1. Nanoparticles were prepared at different mass ratios of PBAE537 to GFP (5:1, 10:1, 20:1, 40:1, 80:1, and 100:1), and the plasmid encapsulation ability of PBAE537 was determined by agar gel electrophoresis. When the mass ratio of PBAE537 to GFP reached 40:1, most of the plasmids were encapsulated by PBAE537, and only a few plasmids migrated. When the mass ratio was increased to 60:1, the plasmid was almost completely encapsulated by PBAE537 and remained in the spot pores of the agarose gel without migration (Figure 2A). The dynamic light scattering particle size range shows that the NPs and different mass ratios were 146.1–354.5 nm, and the electric potential range was 14.5–30.6 mV (Figures 2B,C). The NP size was the minimum when the mass ratio was 40:1, suggesting that the interaction between PBAE537 and the GFP plasmid was closest to this mass ratio. Transmission electron microscopy (TEM) showed that the shape of NPs composed of PBAE537 and GFP was spherical and uniform (Supplementary Figures S1A,B). These results indicate that our synthesized material PBAE537 can wrap nucleic acid to form nanoparticles.

### Uptake of Nanoparticles *in vitro* and *in vivo*

**FIGURE 3:**
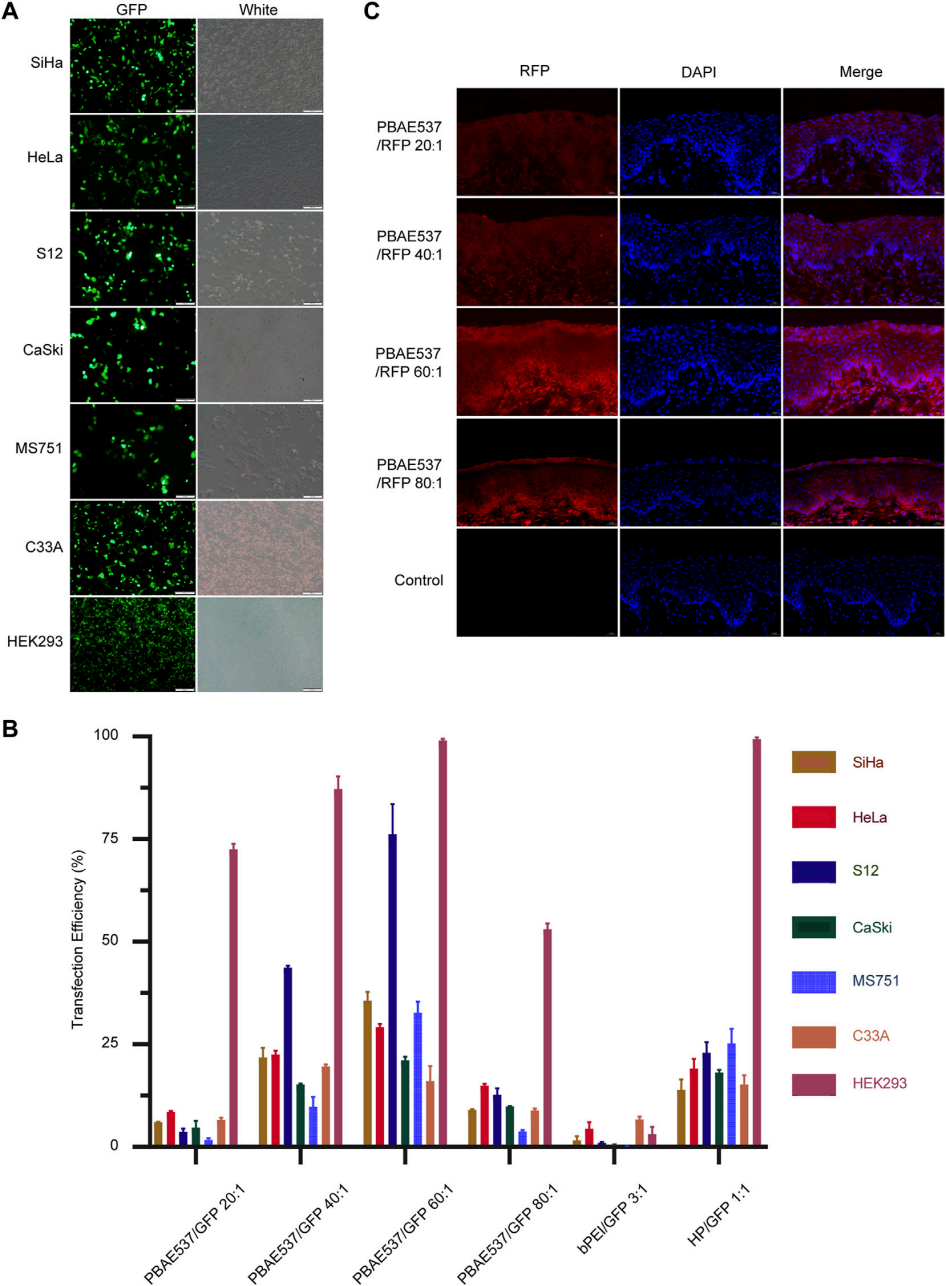
Efficiency of NPs taken up by cell lines and the mouse cervix. **(A)** Representative images of green fluorescent protein expression in SiHa, HeLa, S12, CaSki, MS751, C33A, and HEK293 cells after 72 h of nanoparticle (PBAE537/GFP 60:1) treatment. **(B)** Flow cytometry was used to determine the efficiency of nanoparticles transfected with PBAE537/GFP at different weight ratios (20:1, 40:1, 60:1, and, 80:1) and HP/GFP (1:1) for the expression of the green fluorescent protein. bPEI is a cationic polymer with transfection ability used as a control. **(C)** Representative images of red fluorescent protein expression in the cervix of the C57BL/6 mice treated with nanoparticles composed of different weight ratios of PBAE537 to RFP (10 μg RFP, once daily, for 3 days) compared with the control cervix treated with sodium acetate alone without nanoparticles. Scale, 20 μm.

The efficacy of gene therapy depends on the uptake rate of nanoparticles by targeted cells and the expression rate of delivered genes. *In vitro*, nanoparticles formed by GFP plasmids and PBAE537 were transfected into seven cell lines (SiHa, HeLa, S12, CaSki, MS751, C33A, and HEK293). Transfection efficiency was evaluated by fluorescence microscopy and flow cytometry (Figures 3A,B, Supplementary Figure S5). The transfection efficiency of nanoparticles with the same mass ratio was different in each cell line. In general, the transfection efficiency was highest when the mass ratio of PBAE537 to GFP was 60:1. In some cell lines, such as SiHa, HeLa, S12, and MS751, the efficiency was even higher than that of bPEI (a cationic polymer with transfection ability) and the commercial transfection reagent HP. Transmission electron microscopy imaging of HEK293 cells transfected with nanoparticles revealed the presence of nanoparticles in cytoplasmic vesicles, indicating successful uptake of our nanoparticles into the cells (Supplementary Figure S2).

*In vivo*, nanoparticles composed of PBAE537 and red fluorescent protein (RFP) plasmid were injected vaginally into female C57BL/6 mice, and the uptake of the nanoparticles *in vivo* was assessed by the intensity of red fluorescence **(** Figure 3C **)**. To explore the experimental conditions to achieve the highest transfection efficiency *in vivo*, we adjusted the following conditions: the mass ratio of PBAE537 to GFP, the dose of transfected plasmid, and the detection time point after treatment **(** Supplementary Figure S3 **)**. The results showed that the transfection efficiency was the highest when the mass ratio was 60:1, the plasmid dose was 10 μg every 3 days, and detection occurred on the sixth day after treatment. The expression of fluorescent protein suggested that PBAE537 can efficiently deliver nucleic acid *in vivo* and *in vitro*.

### Toxicity Test of Nanoparticles

**FIGURE 4:**
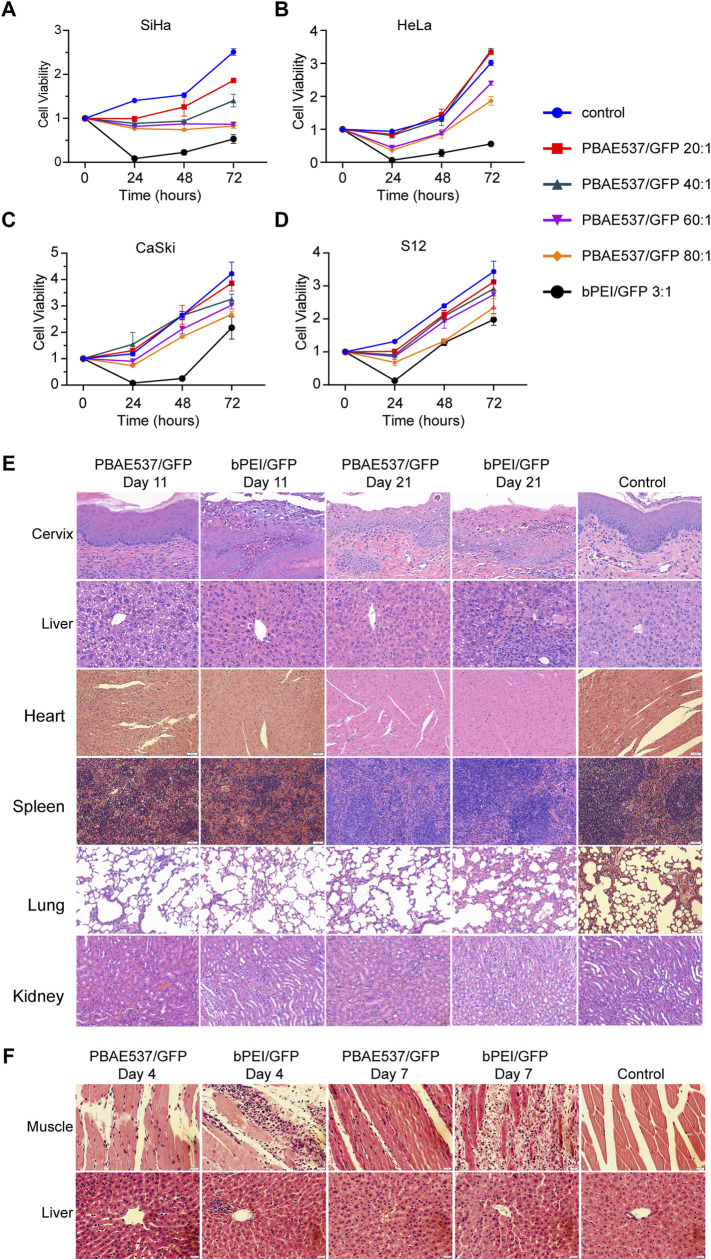
Toxicity analysis of nanoparticles in cell lines and mouse organs. **(A–D)** The toxicity of PBAE537/GFP nanoparticles with different mass ratios (20:1, 40:1, 60:1, and 80:1) was detected in **(A)** SiHa, **(B)** HeLa, **(C)** S12, and **(D)** CaSki cells. bPEI/GFP (3:1) was used as a positive control. The time points of cytotoxicity were 24, 48, and 72 h after transfection. Each time point represents the mean ± SEM (*n* = 4). **(E)** HE staining of paraffin sections of cervical and liver tissues of mice that received continuous vaginal injections of PBAE537/GFP (60:1) and bPEI GFP (3:1) for 10 and 20 days. Mice were injected intravaginally with 10 μL nanoparticles containing 10 μg plasmids. Vaginal treatment was performed once daily for 10 or 20 days. Significant damage was observed only in the bPEI group, but less in the PBAE537 group. **(F)** Nanoparticles composed of PBAE537/GFP (60:1) and bPEI/GFP (3:1) were injected into the thigh muscles of mice for 3 consecutive days. Representative images of HE staining in paraffin sections of thigh muscle tissue and liver tissue of mice at 4 and 7 days after the initial injection. One hundred microliters of plasmids containing 100 μg nanoparticles were injected into the thigh muscles of mice every day. Scale, 20 μm.

To evaluate the toxicity of nanoparticles, we transfected SiHa, HeLa, CaSki, and S12 cells with nanoparticles at PBAE537/GFP mass ratios of 20:1, 40:1, 60:1, and 80:1. Cell viability was detected by CCK-8 assays at 24, 48, and 72 h after transfection **(** Figures 4A–D **)**. When the mass ratio of PBAE537 to GFP was as high as 80:1, growth inhibition was most significant but less than that in the positive control group (bPEI/GFP 3:1). The PBAE537 showed low cytotoxicity *in vitro*.

To further detect the toxicity of nanoparticles *in vivo*, we reviewed the literature (Anderson et al., 2004) and conducted maximum dose toxicity tests with 200 and 300 μg plasmids for vaginal administration and intra-tumoral administration, respectively. Nanoparticles consisting of PBAE537 or bPEI coated with 100 μg of GFP were injected into the vaginas of C57BL/6 mice daily for 20 days, and toxicity was detected on the 11th and 21st days after initiation of vaginal administration. Compared with the untreated control group, the bPEI/GFP treatment group exhibited significant increases in the number of inflammatory necroses in the cervix and pyknosis of the liver nucleus, while no similar changes were observed in the nanoparticle (PBAE546/GFP 60:1)-treated group **(** Figure 4E **)**. Low toxicity was observed in the heart, spleen, lungs, or kidneys. Nanoparticles consisting of PBAE537 or bPEI coated with 100 μg GFP were injected daily into the thigh muscles of C57BL/6 mice for 3 days, and toxicity was detected on the 4th and 7th days after initiation injection. In the bPEI/GFP treatment group, local injection caused large-scale necrotic inflammatory infiltration of muscular tissue, while no similar changes were observed in the 60:1 group treated with PBAE546/GFP **(** Figure 4F **)**. Low toxicity was observed in other important organs **(** Supplementary Figure S4 **)**. The above data indicate our nanoparticles’ safety and low toxicity, which are necessary characteristics for pharmaceutical use.

### Efficacy of Nanoparticles (Poly (Beta-Amino ester)537/Therapeutic Plasmids) on Cervical Cancer Cells *In Vitro* and *In Vivo*

**FIGURE 5:**
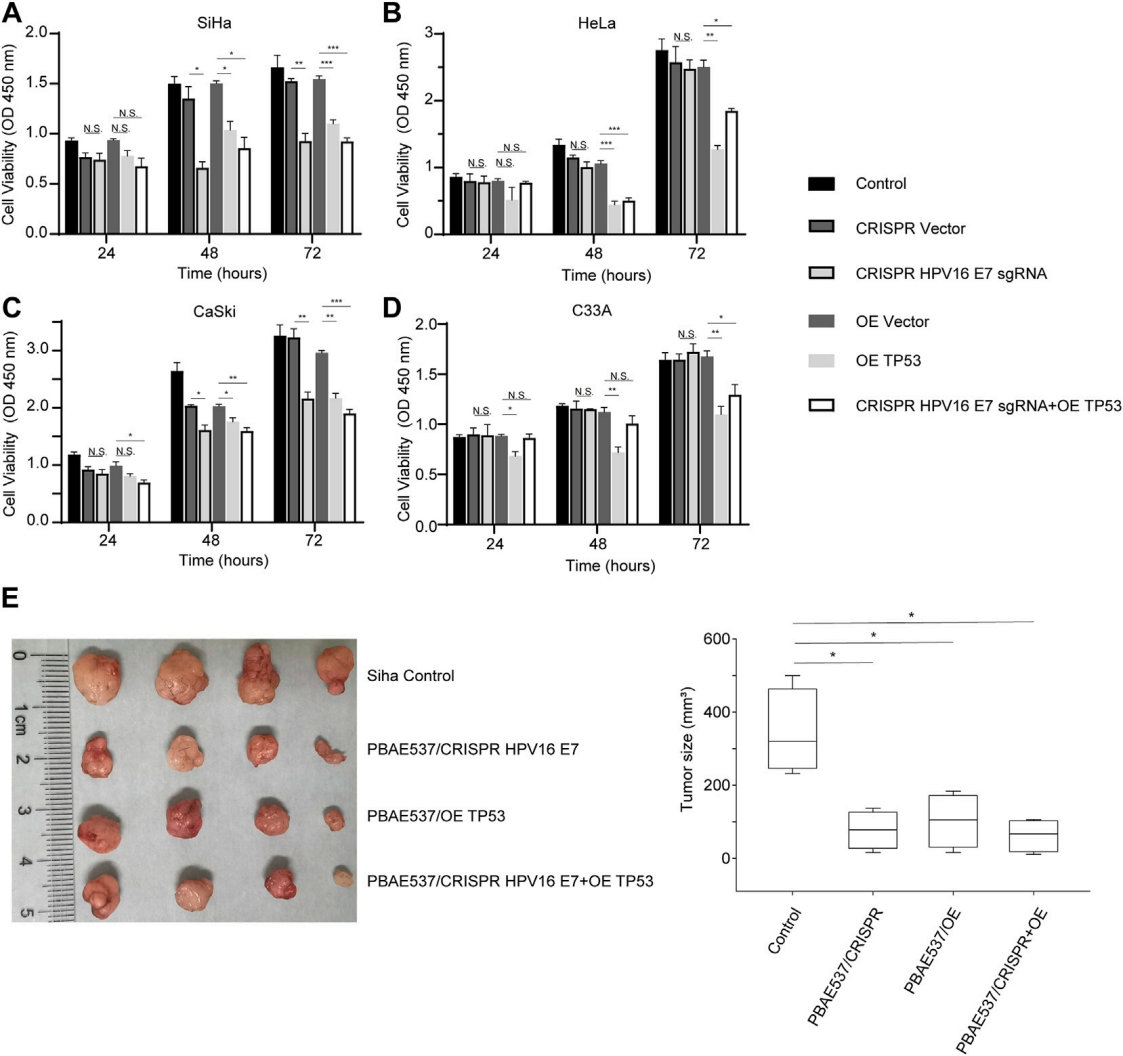
Growth inhibition of cervical cancer cells by PBAE537–therapeutic plasmid polyplex NPs *in vitro* and *in vivo*. **(A–C)** The CCK-8 method was used to detect the cell viability of **(A)** SiHa, **(B)** HeLa, **(C)** S12, and **(D)** CaSki cells at 24, 48, and 72 h after treatment with nanoparticles (PBAE537/plasmid 60:1) composed of PBAE537 and three targeted plasmids (HPV16 *E7* inactivation group, *p53* overexpression group, and 1:1 mixture of the two plasmids group). **(E, F)** SiHa cells were injected subcutaneously into the right hind limb of BALB/c-nu mice. When the transplanted tumor grew to approximately 35 mm^3^, NPs consisting of PBAE537 and the CRISPR/Cas9 plasmid targeting HPV16 *E7* and *p53* were injected intratumorally (60:1). NPs were injected as a volume of 100 µL containing 60 μg of plasmid once every 4 days. **(E)** The subcutaneously formed tumors were photographed, and **(F)** the estimated sizes were measured after treatment with NPs. One-way ANOVA was used for statistical analysis, *: *p* < 0.05, **: *p* < 0.01, ***: *p* < 0.001, N.S.: no significant difference.

Based on the clear understanding of the toxicity of PBAE537, we further studied the therapeutic effect of PBAE537/therapeutic plasmid *in vivo* and *in vitro*. As shown in Figures 5A−D, targeting HPV16 *E7* decreased the viability of HPV16-positive SiHa and CaSki cells but did not significantly inhibit the growth of HPV18-positive HeLa and HPV-negative C33A cells. The *p53* overexpression group and the group with the combination targeting HPV16 *E7* and over-expressing *p53* showed significant growth inhibition in all four cell lines. To observe the inhibitory effect of NPs on tumor growth *in vivo*, SiHa cells were inoculated subcutaneously into nude mice. When xenograft tumors grew to about 35 mm (Klco and Mullighan, 2021), NPs were used for intra-tumoral injection. The grouping of the experiment was the same as that of the *in vitro* experiment, including the single target of HPV16 *E7*, *p53*, and double target of both. Compared with the control group, SiHa subcutaneous tumor growth and volume were significantly reduced in the three groups of targeted therapy mice (Figures 5E,F). Growth inhibition of cervical cancer cell lines and subcutaneous tumors confirmed the therapeutic effect of our PBAE537 nanoparticles.

### Effects of Nanoparticles Targeting HPV16 *E7* and *p53* on Protein Expression in HPV16 Transgenic Mice

**FIGURE 6:**
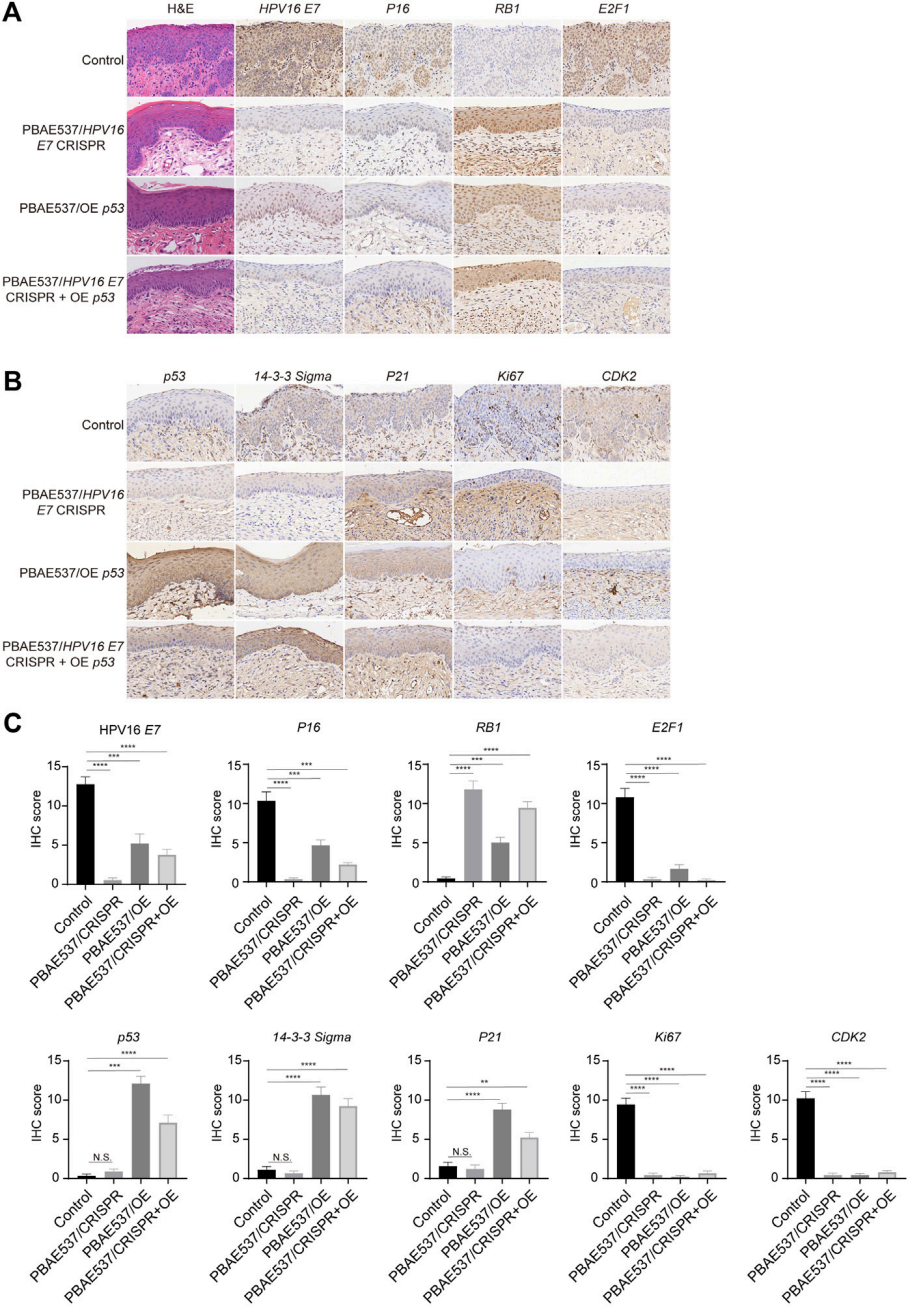
Therapeutic effects of nanoparticles composed of PBAE537 and therapeutic plasmids targeting HPV16 *E* ***7*** and p53 K14-HPV16 transgenic mice. **(A, B)** Representative images of H and E and IHC staining and **(C)** comparison of the cervical epithelium between untreated HPV16 transgenic mice (control group) and three other targeted groups treated with nanoparticles composed of PBAE537 and plasmids (60:1, 10 μg of plasmids per day for 20 days continuously. IHC staining indicators included HPV16 *E* ^*7*^, *P16*, *RB1*, *E2F1*, *p53*, *14-3-3 Sigma*, *P21*, *Ki67*, and *CDK2*. Scale bars, 20 μm. The data represent the mean ± SEM (*n* = 3). One-way ANOVA was used for statistical analysis, *: *p* < 0.05, **: *p* < 0.01, ***: *p* < 0.001, ****: *p* < 0.0001, N.S.: no significant difference.

Encouraged by the above results, we next performed cervical *in situ* therapy with nanoparticles in HPV16 transgenic mice (Medler et al., 2018), a cervical precancerous lesion model. After 20 days of continuous treatment, the malignant phenotype of the cervical epithelium in the mice treated with the three targeted nanoparticles was significantly reversed. The proliferation of epithelial cells was inhibited, the basal cells were arranged neatly, and the nuclear volume was reduced. The cervical epithelial cells of the untreated control mice were hyperplastic. Immunohistochemistry was used to detect the expression levels of HPV16 *E7* and *p53* and related proteins. The expression of the target molecule HPV16 *E7* and the HR-HPV substitute marker *P16* was significantly reduced in all three targeted administration groups, while the expression of the tumor suppressor gene *RB*, which interacts with the *E7* oncoprotein, was restored. The levels of *p53* and its downstream molecules *P21* and *14-3-3 Sigma*, which are responsible for cell cycle regulation, were increased to a certain extent in both targeted activation groups. The expression of the proliferation-related protein *Ki67*, cell cycle-related protein *CDK2*, transcription factor *E2F1*, and apoptosis suppressor gene *Bcl2* was significantly inhibited (Figure 6). These results suggest that our nanoparticles targeting HPV16 *E7* or supplementation with wild-type *p53* can effectively reduce the expression of oncoproteins, restore the expression of suppressed tumor suppressor factors, and affect the related signaling pathways, thereby reversing the malignant phenotype of cervical epithelium in HPV16 transgenic mice.

## Discussion

In this study, we developed the nanomaterial PBAE537, which showed low biotoxicity, degradability, and high transfection efficiency (Figures 1, 2). In addition to the size of the nanoparticles, the electric potential of the nanoparticles and the characteristics of the transfected cells also affect the transfection efficiency. These all affect the ability of nanoparticles to encapsulate and release plasmids. We believe that PBAEs are suitable for vaginal usage because the acidic environment of the vagina is conducive to the stability of PBAEs (Li et al., 2020). Vaginal usage can significantly reduce the amount of PBAEs in the blood and thus significantly reduce side effects such as hemolysis. Toxicity is the greatest challenge in nanomaterial therapy, so we chose to fully evaluate the toxicity of PBAE537 by muscular and vaginal delivery forms. The effects on other organs can be observed after nanoparticles enter the circulatory system by muscle administration. Vaginal delivery has the advantage of mimicking what occurs in clinical trials, although a small fraction of the drug may flow out of the vagina. PBAE537 showed very low biotoxicity (Figure 4), which is very promising for the further clinical transformation of drugs. In addition, PBAEs are highly plastic, and higher efficacy and fewer side effects can be achieved by optimizing their structure (Liu et al., 2019). The delivery efficiency of PBA537 was satisfactory (Figure 3). These optimizations deserve further study.

We selected therapeutic targets. The *E6* and *E7* viral oncogenes encode oncoproteins that disrupt cell cycle regulation and enhance cell proliferation by inactivating the tumor suppressors *p53* and *RB*, respectively (Longworth and Laimins, 2004; Strickland and Vande Pol, 2016; Ghanaat et al., 2021). *P53* inactivation mediated by *E6* or *p53* mutations may be a key step in the development of cervical cancer (Scheffner et al., 1991; Park et al., 1994). Previous studies have used CRISPR/Cas viral vectors to specifically inactivate *E6* or *E7* in tumor cells, but these therapies are limited by the safety of viral vectors and the specificity of oncogene sequences of different HPV types. Their downstream tumor suppressors, such as *p53*, avoid the complexity of the single guide RNA design. Dysfunction of the tumor suppressor *p53* is closely related to insensitivity to treatment and recurrence of many malignant tumors, including cervical cancer.

An increasing number of studies have shown that the recovery of *p53* activity can induce cell cycle arrest and apoptosis, eliminate chemoradiotherapy resistance, and inhibit the growth of tumor cells (Einbond et al., 2021; Granados-López et al., 2021). Therefore, activating wild-type *p53* and restoring *p53* function seems to be an attractive therapeutic strategy (Merlin et al., 2021). Pharmacological activation of the *p53* pathway has been reported for the treatment of skin cancer, neuroblastoma, prostate cancer, and other tumors (Loureiro et al., 2020; Patiño-Morales et al., 2020; Won and Seo, 2020; Zafar et al., 2021). Some studies directly restore *p53* expression to achieve the purpose of tumor treatment (Blandino and Di Agostino, 2018; Kong et al., 2019; Hu et al., 2021). Reactivation of *p53* has also been considered as a potential treatment in cervical cancer (Zhao et al., 2021). Thus, in this study, we chose to focus on the efficacy of restoring *p53* expression and targeted knockout of *E7* in a mouse model of cervical precancerous lesions. The CRISPR/Cas9 recombinant plasmid expressing wild-type *p53* and the overexpression plasmid targeting HPV16 *E7* and their mixtures were delivered by PBAE537.

Nucleic acid delivery by PBAE has been used in many kinds of tumors (Zhang et al., 2020; Qu et al., 2020; Niu et al., 2020; Kaczmarek et al., 2018). For example, it was reported that the transfection efficiency of PBAE in B16-F10 cells was 65% (Routkevitch et al., 2020). Our PBAE537 showed comparable transfection efficiency and low toxicity to previous studies, both *in vitro* and *in vivo*. In this study, growth inhibition of cervical cancer cell lines (SiHa (HPV16 positive), HeLa (HPV18 positive), CaSki (HPV16 positive, HPV18 positive), and C33A (HPV negative)) and subcutaneous tumors confirmed the therapeutic effect of our PBAE537 nanoparticles (Figure 5). Restoration of *p53* expression in cervical vaginal epithelium of HPV transgenic mice reversed the phenotype of CIN (Figure 6). Restored *p53* expression increases apoptosis of epithelial cells, and when apoptosis increases, E7 and Rb expression may be affected. We hypothesized that *p53* overexpression may restore RB expression through some pathways. Previous reports have confirmed the cross-talk between *p53* and Rb/E2F signaling mechanisms (Rajasekaran et al., 2017). We can see that *E7* is still expressed in the basal region. The expression of *E7* is derived from exogenous HPV and is relatively independent, so it remains expressed in the basal region even with increased apoptosis.

Our work suggests that the restoration of *p53* expression and the inactivation of HPV16 *E7* are essential for blocking the development of cervical cancer. Restoration of *p53* activity can be extended to a variety of tumors, and the PBAE537 delivery vector can also be used for the treatment of other genes and tumors. Restoration of *p53* expression can also be applied to other tumors caused by *p53* inactivation, such as lung cancer, hematological malignancies, etc., becoming a promising broad-spectrum targeted anticancer drug.

## Data Availability

The original contributions presented in the study are included in the article/Supplementary Material, further inquiries can be directed to the corresponding authors.
