# Supplementary material for: Co-Delivery of p53 Restored and E7 Targeted Nucleic Acids by Poly (Beta-Amino Ester) Complex Nanoparticles for the Treatment of HPV Related Cervical Lesions
Source: Front Pharmacol. 2022 Feb 4;13:826771. doi: 10.3389/fphar.2022.826771 (PMC8855959; doi:10.3389/fphar.2022.826771)
Supplement: Supplementary file 1 [file DataSheet1.docx]

**Restored of p53 and targeted to E7 nucleic acids by poly (beta-amino ester) complex nanoparticles for the treatment of HPV related cervical lesions**

**Supplemental Figures:**

**Supplemental Figure 1: Characterization of NPs.** (A-B) TME imaging of PBAE537/GFP NPs (weight ratio 60:1). (A) Scale bar, 1 μm. (B) Scale bar, 500 nm.


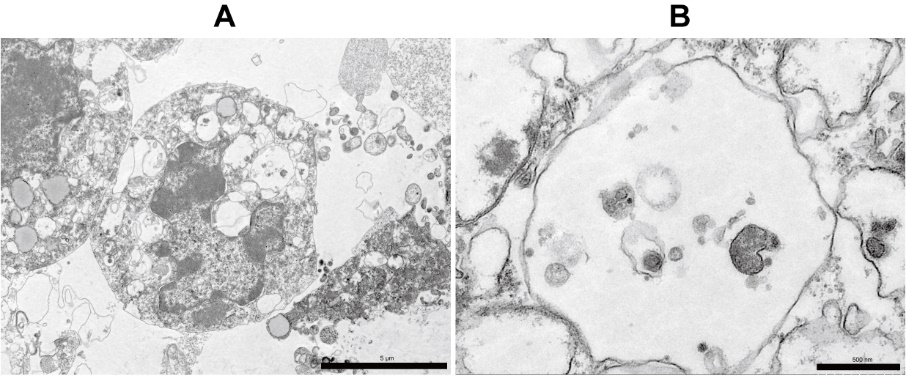


**Supplemental Figure 2: Transmission electron microscopy (TEM) imaging of 293 cells transfected with PBAE537/GFP NPs.** NPs were in cytoplasmic vesicles, indicating endocytosis. (A) Scale bar, 5 μm. (B) Scale bar, 500 nm.


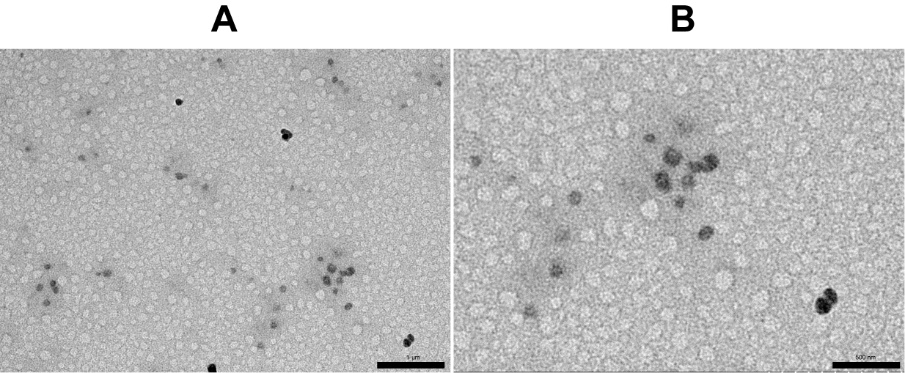


**Supplemental Figure 3: Comparison of the uptake of NPs with different time points after treatment and doses of RFP in the cervixes of C57BL/6 female mice.** Representative fluorescence images of the uterine cervixes of C57BL/6 mice treated (A) at different time points after treatment (PBAE537/RFP, weight ratio 60:1, 10 μg RFP once a day for 3 days), and (B) with different doses of RFP (PBAE537/RFP, weight ratio 60:1, once a day for 3 days).


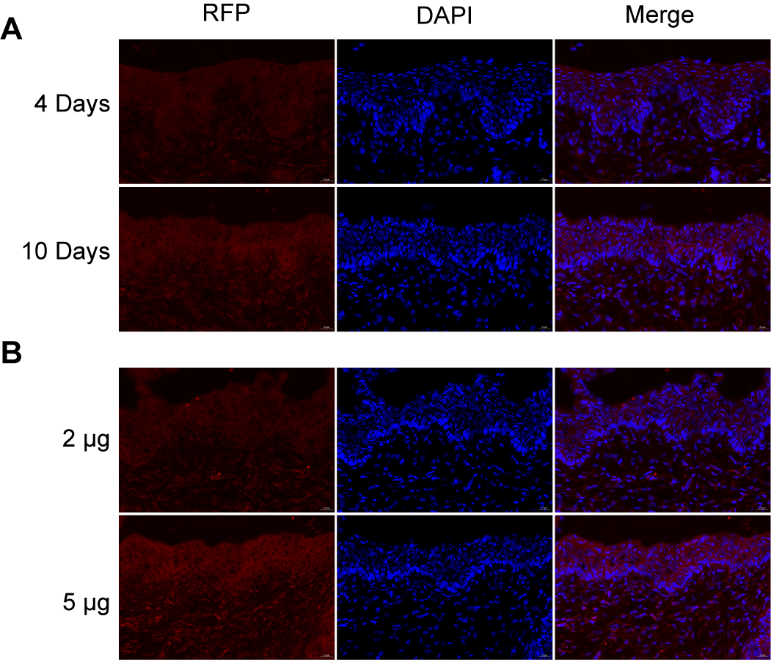


**Supplemental Figure 4: Toxicity of NPs in the organs of mice whose thigh muscles were injected with NPs.** Representative H&E staining images of the hearts, spleens, lungs and kidneys of C57BL/6 female mice whose thigh muscles were injected with NPs. The NPs consisted of 537/GFP (weight ratio 60:1) or bPEI/GFP (weight ratio 3:1). NPs carrying 100 µg of the plasmid were injected into the thigh muscles of the once per day for 3 days, and the organs were harvested on the 4th and 7th days after the initial administration. Scale bars, 50 µm.


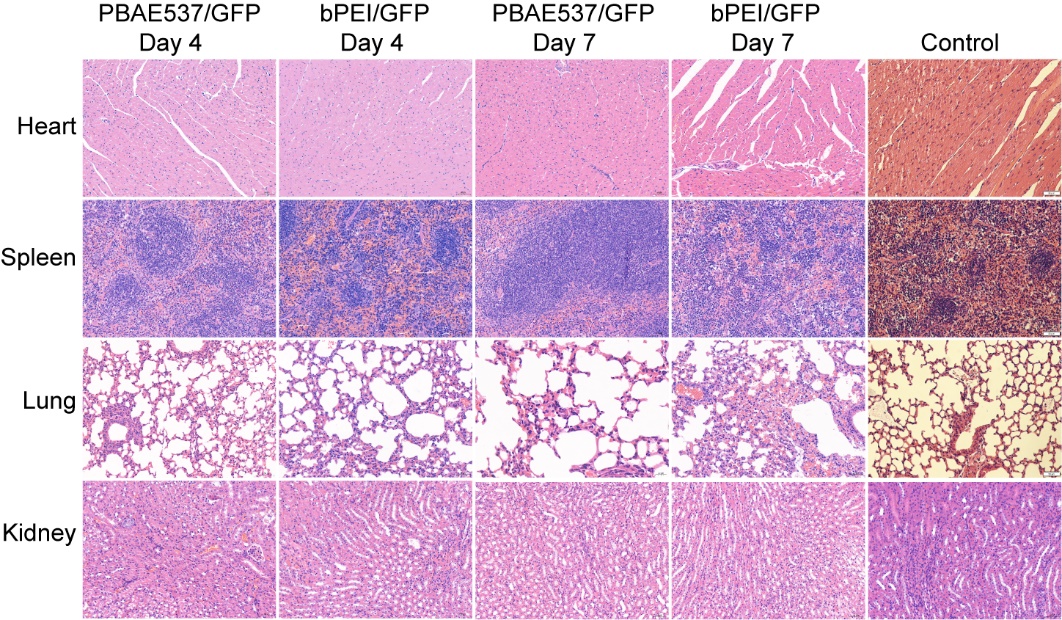


**Supplemental Figure 5: Cas9 expression in western blot with the polymeric nanoparticles in SiHa.** Cas9 was detected by western blot in SiHa cells transfected with CRISPR-loaded nanoparticles. GAPDH was used as a loading control.

**
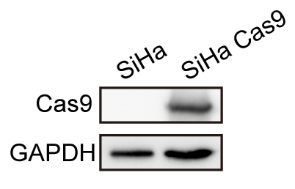
**
